#  Comparison of Serum Zinc Levels among Children with Simple Febrile Seizure and Control Group: A Systematic Review 

**Published:** 2015

**Authors:** Mohammad Mehdi NASEHI, Roya SAKHAEI, Mahmood MOOSAZADEH, Maryam ALIRAMZANY

**Affiliations:** 1 Pediatric Neurology Department, Mofid Hospital, Shahid Beheshti University of Medical Sciences, Tehran, Iran; 2 Faculty of health, Kerman University of Medical Sciences, Kerman, Iran,; 3 Health Sciences Research Center, School of Health, Mazandaran University of Medical Sciences, Sari, Iran; 4 Research Center for Health Services Management, Institute for Futures Studies in Health, Kerman University Of Medical Sciences, Kerman, Iran

**Keywords:** Febrile seizure, Febrile convulsion, Zinc, Trace elements, Systematic review

## Abstract

**Objective:**

Several factors are involved in the etiology of febrile seizure (FS), among them is zinc (Zn), which has been discussed in various studies. The present systematic review compares Zn levels in children with FS and a control group.

**Materials & Methods:**

We searched keywords of febrile seizure, febrile convulsion, children, childhood, fever, trace elements, risk factor, predisposing, zinc, Zn, and epilepsy in the following databases: SCOPUS, PubMed, and Google Scholar. The quality of research papers was assessed using a checklist. Data was extracted from primary studies based on demographic variables and amounts of Zn in case and control groups.

**Results:**

Twenty primary studies were entered in the present study. Of which, eighteen studies, reported that Zn serum levels were significantly lower in the case group (patients with FS) than the control group.

**Conclusion:**

The present systematic review indicated that Zn is one factor for predicting FS. A low level of this element among children can be regarded as a contributing factor for FS, a conclusion with a high consensus among different studies carried out in different parts of the world.

## Introduction

Febrile seizure (FS) is a common neurological disorder among children, which has been observed in 2–5% of cases. FS occurs with fever (temperature degree greater than 38°C) and without symptoms of central nerve infection, severe electrolytic disorder, or a specifically defined cause (1-6). Considering the high incidence of feverish disease among children under 5-years of age, every bacterial or viral disease may cause FS, Thus, it is expected that each FS concerns parents and causes different problems (7-8). Previous studies (1-2, 4-6) have shown that fever related seizures are the most common convulsions among children aged from 6-month–5- years. In a case control study done on 100 children (50 cases and 50 controls aged 6-months–6-years of age) admitted in a referral hospital in northern Iran, the mean serum Zinc was significantly lower in the case group (children with simple FS) compared to the control group (9). It is notable that 50% of children below one year with FS experience relapsed, so it is necessary to pay special attention to this issue and consider social-mental effects, cognitive disabilities, occupation of hospital bed, increased length of hospitalization, and costs of treatment in these patients (10). 

Previous studies have shown that refrigerants do not prevent FS; therefore, effective factors of FS are investigated by many researchers (1-2, 11-12). However, the mechanism of FS is still unknown. The literature (1- 2, 4-6, 9-11) has suggested different factors responsible for FS, some of them are known as certain factors, while others are probable factors. Various theories have been posed about the role of neurotransmitters and trace elements of serum and cerebrospinal fluid as pathogenesis of FS; also, a great deal of research has been done (1-2, 4-6, 9-11). The trace elements are performing different functions in the body, including their association with FS in children. One of these elements is Zn and has a serum level correlation with FS has been widely investigated (12-19). 

Initial electronic search on the correlation of Zn serum level and FS in children has indicated that various studies have been carried out in this field with some conflicting results, which confusing reader. Therefore, it is necessary to combine them to lower these conflicts and to set clearer research priorities for future studies. 

Systematic reviews are of the most important studies to extract and gather statistical data from several studies (13). In a systematic review, research papers and other documents are searched according to a predefined criterion. Such studies are highly reliable, due to observing rigorous methods and the use of this technique is widespread to study various phenomena. 

Since no secondary study has yet been carried out to examine the correlation of Zn serum levels and FS, we intend to extract, gather, and present all available published data and research in English to enhance evidence based decision making in this area. In addition, we will conduct an analytic study based on the documents. 

## Materials & Methods

This systematic review was done to determine the correlation of Zn serum levels and FS in children. 

**Search strategy**

To retrieve studies, using the English databases of Scopus, PubMed and Google Scholar to search using keywords for febrile seizure, febrile convulsion, children, epilepsy, Zinc, Zn, predisposing, risk factor, trace elements, childhood, fever, along with “and” and “or” operators (Articles which were published up to 5/ 30/ 2013). We reviewed the reference list of published studies to increase the sensitivity and to select more studies, which we could not retrieve from databases. One of the authors who did not participate in the search assessed the search randomly. Ultimately, it was specified that no study have been missed. 

**Selection of studies**

Full texts or abstracts of all papers, documents, and reports were extracted. After reviewing the titles of documents, the name(s) of the author(s), and year of publication, any duplications were removed. It is worth mentioning that to prevent bias in the transversely reprinting data, scholars reviewed data to identify and to eliminate duplicated research. After that, we studied the articles carefully to select relevant articles and exclude non-relevant ones. 

**Quality assessment**

After determining relevant studies based on title and content, a review of 2 articles “Guidelines to provide studies report based on strobe statements”(14) and reviewing “Methodological quality of systematic review studies” (15) as well as experiences of researchers, some questions were designed to assess the quality of the studies. These questions covered aspects like, type of study (case-control), sample size, age group (less than 72 mounts), sampling method, study objectives, observed population, definition of inclusion and exclusion criteria to initial studies, method of matching samples, analysis method, presentation of findings, and providing the results based on objectives. Each question had one point; every study that received at least 8 points was included in this review (13). Since we did not want to do meta-analysis and to use quality ratings of studies as an independent variable in the meta-regression model, we neglected things like carrying out research by an organization or university, doing research by a well-known scholar, and publishing the article in a prestigious journal with a high impact factor. 

**Data Extraction**

Authors extracted data for each primary study based on article title, first author, year of publication, age group, matched variables, sample size disaggregated by case and control groups, the place of study, the amount of Zn prevalent in case and control groups, control group definition, unit of measurement for Zn serum levels, and the measurement methods of Zn serum levels. 

**Inclusion criteria**

Any case-control study in English that reviewed the correlation of Zn serum levels and FS in children was used for this study after an initial evaluation process and to obtain essential scores for analysis. 

**Exclusion criteria**

After an initial search with keywords, some non-relevant papers were excluded by reviewing the title, some from the abstract, and others by reviewing the full text. Then, the quality of the remaining papers was evaluated using a checklist and the studies that had received a score less than 8 were set aside. In addition, studies with observed population of children with a record of FS, epilepsy, neurological impairment doubt about brain infection, malnutrition, and complex convulsions were omitted. It is noteworthy that articles with only an English abstract were included, because they contained all information required for this study. Due to high heterogeneity between the results of primary studies, the use of different tools and methods in the testing samples, variations in normal values of variables in earlier studies, and the direct and inverse effect of Zn serum levels on FS in primary studies and the conditions were not suitable for conducting meta-analysis. 

## Results

**Fig 1:**
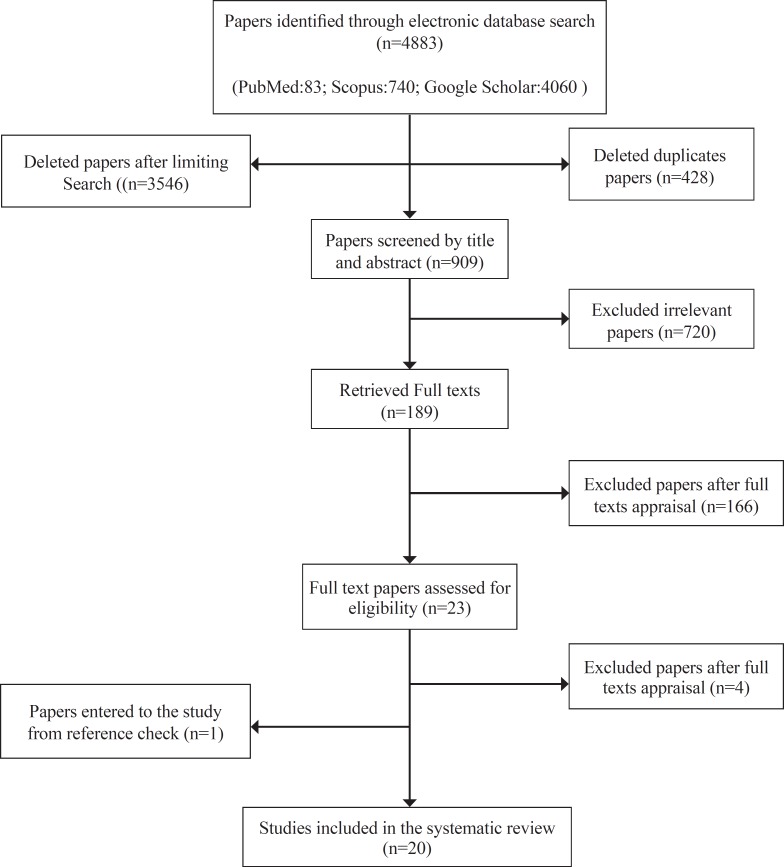
Process for papers search and review flowchart for selection of primary studies

Using relevant keywords and applying maximum sensitivity, 4,883 articles were identified searching various English databases. We omitted 3,546 articles after limiting keywords and 428 articles were removed due to overlapping among databases. Reviewing the titles and abstracts of 909 primary studies, 720 irrelevant primary studies were excluded, which left 189 articles that were chosen for a full text review. After reviewing the full text of articles and omitting more non-relevant ones, 23 articles were assessed using the checklist of quality assessment, and a further 4 articles (16-19) were eliminated due to not achieving the required score, i.e. less than 8. Searching the references, one article (20) was added to the study; ultimately, 20 relevant studies (20, 11, 6, 2, 1, 21-35) were entered into this systematic review (Figure 1). 

Publication year of these primary studies were entered into this systematic review and varied from 1996 (Gunduz, Turkey) to 2012 (Kafada, Turkey; Lee, South Korea; Mattheas, Nigeria). Regarding place of study, these studies have been carried out in Iran, Turkey, Indonesia, Bangladesh, South Korea, India, and Nigeria (Table 1). 

Observed age group was from 3–72 months and in 8 primary studies the methods for matching control and case groups was mentioned. The control group in most primary studies (15 cases) was comprised of feverish children without convulsion. The measurement method for Zn serum levels in most of studies was atomic absorption spectrometry (Table 1). 

In 12 studies, treatment and control group were equal; in 7 studies, the control group size was smaller than the case group size; and in one study, the control group was greater than the case group (Table 1). 

Table 1 shows 18 initial studies that the Zn serum levels in the case group (patients with FS) was significantly less than for the control group. 

Cho (South Korea, YEAR) indicated that Zn serums levels in the case group were lower than for the control group, but the observed difference was not significant. It is important to note that in just in one study (Kafadar, Turkey) that Zn serum levels for the case group were more than for the control group, but the difference was not significant (P>0.05).

## Discussion

In this systematic review, we studied the relationship between Zn serum levels and FS using 20 qualified studies and used for this study out of an initial set of 4,883 documents. This study showed that Zn serum levels in the case group were significantly lower than for the control group. 

In a case-control study by Ehsanipoor et al (21), 92 children aged 6 months–5 years of age were classified into 3 groups of children with FS (34 persons), children with fever without convulsion (40 persons), and children with convulsion without fever (18 persons). Mean Zn serum levels for these groups was 76±24.36, 90.12±14.63, 94±17.39, respectively. There was a significant difference between Zn serum levels in the three groups and children with FS, Zinc serum levels were significantly lower than for the other two groups. Margareta et al (24) (Indonesia) indicated that mean zinc serum levels of the case group was less than for the control group. Table 2 compares Zn serum levels in two groups of patients with FS and a control group in different countries to show that serum levels of Zn in patients with FS was lower than for normal children or children with fever but without convulsions. As these studies have shown, there is a consensus on the effect of Zn serum levels on FS.

**Table 1:** The Result of Serum Zn Levels in Children with Simple FS and the Control Group in Primary Studies

**Row**	**First author, publication year,**	**Local study**	**Sample size**			**Age** **group(months)**	**Matching**	**Serum level**			**Definition of control**	**Method of measure serum**	**Unit of measure serum**
			**Total**	**Case**	**Control**			**Case**	**Control**	**P**			
1	Amiri, 2010(6)	Iran	60	30	30	<60	Age, sex	66.13	107.87	<0.0001	fever without convulsion	AAS	μg/dl
2	Ehsanipour, 2009(21)	Iran	74	34	40	6 to 60	Age, sex	76.82	90.12	<0.006	fever without convulsion	AAS	mg/l
3	Heydarian, 2010(1)	Iran	60	30	30	6 to 72	not declared	663.7	758.33	<0.001	fever without convulsion	AAS	mg/l
4	Kafadar, 2012(22)	Turkey	68	45	23	6 to 72	Age, sex	110.49	107.12	>0.05	fever without convulsion	AAS	μg/dl
5	Lee, 2012(23)	Korea	288	248	40	<60	not declared	60.5	68.9	<0.001	afebrile seizures	not declared	μg/dl
6	Margaretha, 2010(24)	Indonesia	50	25	25	6 to 60	Age, gender, nutritional status	8.83	13.72	<0.05	fever without convulsion	not declared	μmol/l
7	Mollah, 2008(25)	Bangladesh	80	50	30	<60	not declared	464	749.33	<0.001	non-seizure febrile	GF-AAS	*μ*g/L
8	Mahyar, 2008(2)	Iran	104	52	52	9 to 60	age, sex, weight, height, head circumference	62.84	85.70	< 0.05	healthy children	AAS	μg/dl
9	Mollah, 2002(26)	Bangladesh	72	42	30	5 to 72	not declared	40.19	74.98	<0.001	fever without convulsion	AAS	mgm/L
10	Modarresi, 2011(27)	Iran	30	30	30	9 to 60	Age,	93.39	130.54	<0.001	fever without convulsion	GF-AAS	mcg/dl
11	Tutuncuoglu, 2001(28)	Turkey	35	15	20	4 t0 48	not declared	1.23	2.08	<0.05	children with meningeal irritation and fever	ICP-AES	mg/L
12	Talebian, 2009(29)	Iran	120	60	60	3 to 72	not declared	116.28	146	0.003	fever without convulsion	Centerior kit and BT3000	mg / dl
13	Gunduz, 1996(30)	Turkey	60	40	20	6 to 60	not declared	0.7	1.07	<0.001	fever without convulsion	AAS	μg/dl
14	Cho, 1999(31)	Korea	22	11	11	<60	not declared	90.38	97.16	>0.05	fever without convulsion	not declared	mmol/L
15	Cho, 2004(32)	Korea	93	53	40	<60	not declared	74.71	87.03	<0.05	aseptic meningitis	not declared	microgram/dL
16	Ganesh, 2011(33)	India	45	23	22	<60	not declared	638·1	939·9	<0·05	fever without convulsion	not declared	ug/L
17	Ganesh, 2008(34)	India	76	38	38	3 to 60	not declared	32.17	87.6	<0.001	fever without convulsion	not declared	microg/dL
18	Okposio, 2012(35)	Nigerian	180	90	90	<60	Age, sex	58.7	90.3	<0.001	fever without convulsion	AAS	μg/dL
19	Palliana, 2008(20)	India	125	75	50	6 to 72	Age, sex	81.84	90.38	<0.001	fever without convulsion	AAS	mcg/dl
20	Sadegzadeh ,2011(11)	Iran	79	39	40	6 to 60	not declared	75.67	87.58	<0.001	fever without convulsion	AAS	microgram/dL

AAS= atomic absorption spectrometry, GF-AAS= Graphite Furnace Atomic Absorbance Spectrophotometering, ICP-AES= inductively coupled plasma–atomic emission spectrometry

Kafadar (Turkey, YEAR) and Cho (South Korea, YEAR) indicated that the differences of Zn serum levels between the case and control groups were not statistically significant, but it might also be due to small sample sizes of these studies. Both (31, 22), had a control group with 23 and 11 persons respectively, which was half the case group. 

Zn is an essential element for functions of body’s systems especially the central nervous system. Generally, theories to explain a decrease in Zn serum levels in children with FS are as follows: Zn is a regulator of activity for glutamic acid decarboxylase that has a role in the synthesis of gamma- aminobutyric acid (GABA), which is an important neurotransmitter and inhibits the GABA-A receptors in dentate granular cells available in epileptic tissue. The decrease of Zn in the blood and central nervous system, makes N-Mothy1-D-Asparate receptors active, eliminates inhibitory effect of GABA, and, hence, causes seizures/convulsions. It is important to note that in some studies, the changes in cytokine levels, Zn levels, and cerebrospinal fluid have been mentioned as the pathogenesis of FS (11, 26, 36-37) 

One of the limitation of this review is lack of conditions for conducting a meta-analysis, which was due to the heterogeneity of primary studies and their structures to conduct the study, measurement methods of Zn serum levels, selection of control group, intended parameters to report the findings, and matching the case and control groups. Another limitation of this study is in the results of primary studies that have not included matching of variables in the case and control groups. Zn serum levels are different for male and female children at different ages. For example, a study (21) showed that Zn serum levels in girls was lower than for boys (84.12 VS 87.56 mg/l). Additionally, there is a statistically significant correlation between the age of studied person and the value of Zn serum levels. Zn is also influenced by factors such as hemolysis, malnutrition, dehydration, fever, and infection, which makes the selection of a group for comparison crucial. 

**In conclusion, **the present systematic review indicated that Zn is a predictor of FS. A low level of this element among children can be regarded as a contributing factor for FS, a conclusion with a high consensus among different studies carried out in different parts of the world. 

Given the known role of genetic factors and a history of convulsion in the family in the etiology of FS, it is recommended that Zn be prescribed for high-risk children. As body temperature increases can decrease serum Zn levels, feverish children (children with fever) should play the role as a control group in future studies to minimize the reduction error of Zn serum levels caused by fever. It is important to note that matching in terms of sex, age, nutritional status, dehydration, fever, infection, weight, height, and head circumstance should be considered in the design of future studies. 
